# Segmental layered incision and circumferential endoscopic dissection technique of endoscopic stricturotomy for safe, staged endoscopic dissection of complex luminal stricture

**DOI:** 10.1016/j.vgie.2025.08.007

**Published:** 2025-09-02

**Authors:** Partha Pal, Rajesh Gupta, Manu Tandan, D. Nageshwar Reddy

**Affiliations:** Medical Gastroenterology, Asian Institute of Gastroenterology, Hyderabad, India

## Abstract

**Background and Aims:**

Management of tight, fibrotic colonic strictures remains a challenge because of the risk of bleeding or perforation with conventional radial stricturotomy. We describe a novel segmental layered incision and circumferential endoscopic dissection (SLICE) technique, inspired by endoscopic submucosal dissection, designed to improve precision and safety during endoscopic stricture therapy.

**Methods:**

A 32-year-old woman with a 2.5-cm fibrotic colonic stricture at the splenic flexure, presumed secondary to tuberculosis, presented with subacute obstruction. Over 3 consecutive days, SLICE was performed using an insulated-tip knife nano (Olympus, Tokyo, Japan) in a staged manner, progressively dissecting the stricture wall in circumferential, shallow layers. Electrosurgical settings used were ENDO CUT I (effect 3, duration 1, interval 3) on the VIO 300 D unit (Erbe Elektromedizin GmbH, Tübingen, Germany).

**Results:**

The stricture was safely traversed with a pediatric colonoscope after staged dissection totaling 5 hours. No perforation or bleeding occurred. At 6-month follow-up, intestinal ultrasound confirmed resolution of obstruction, and endoscopy showed only mild mucosal reapproximation with sustained luminal patency. A clip-based endoscopic stricturoplasty was performed to prevent reapproximation.

**Conclusions:**

SLICE offers a reproducible, layered approach for complex fibrotic strictures and may reduce the risk of adverse events in high-risk locations. Long-term validation and procedural standardization are warranted.

## Case description

A 32-year-old woman with a history of abdominal pain presented with progressive symptoms of intermittent abdominal pain, bloating, and subacute intestinal obstruction. Cross-sectional imaging suggested a short-segment stricture at the splenic flexure with a small organized collection in the subhepatic area. Colonoscopy confirmed a long, tight, fibrotic stricture at the splenic flexure. Biopsy samples taken while the patient was at another institution of care showed granulomatous inflammation. The findings of a tuberculin skin test were strongly positive. She was prescribed antitubercular therapy, which she discontinued after 1 month.

**Figure 1:**
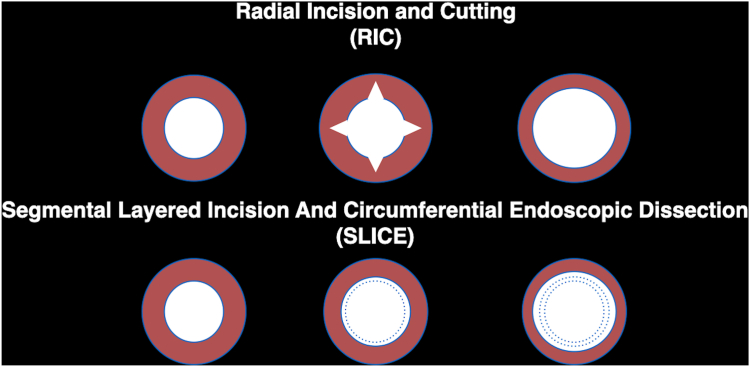
Schematic diagram showing differences between radial incion and cutting (RIC) and segmental layered incision and circumferential endoscopic dissection (SLICE) techniques of endoscopic stricturotomy.

**Figure 2:**
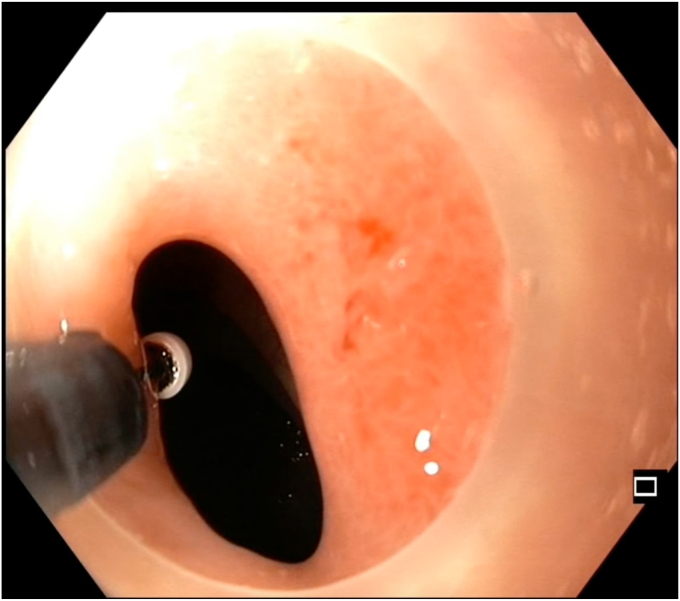
Preprocedural colonoscopic view showing a tight, long fibrotic stricture at the splenic flexure, with a narrowed lumen and insulated-tip knife (Olympus, Tokyo, Japan) introduced to begin dissection.

Given the suspected tubercular origin and the patient's reluctance to undergo surgical diversion, we proposed a staged endoscopic approach using the segmental layered incision and circumferential endoscopic dissection (SLICE) technique (Fig. 1). After shared decision-making and anesthetic clearance, the procedure was initiated with the patient under propofol sedation using a therapeutic endoscope (GIF-HQ190; Olympus, Tokyo, Japan) with a transparent distal cap (D-201-11804; Olympus) (Fig. 2).

**Figure 3:**
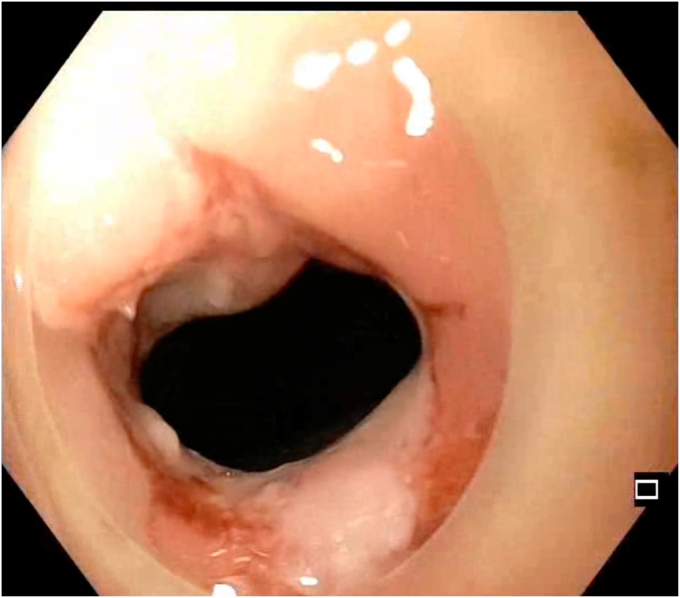
After first session of endoscopic stricturotomy.

**Figure 4:**
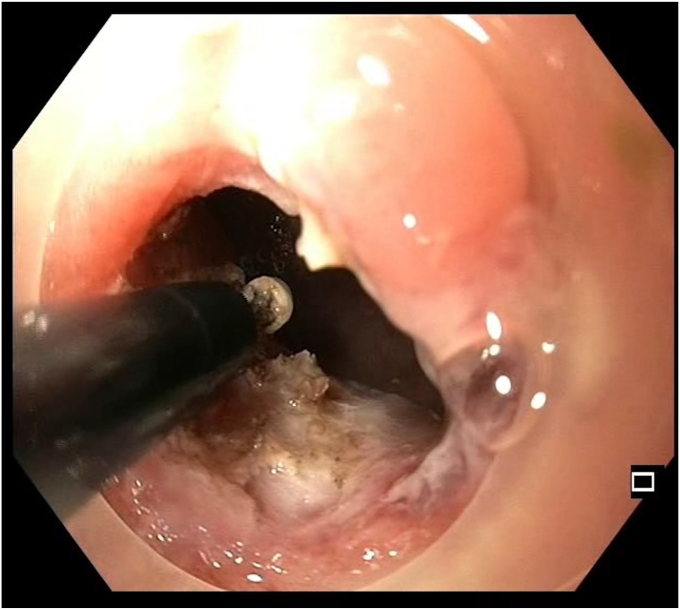
Midprocedural view showing layered circumferential dissection; progressive separation of fibrotic bands occurs.

**Figure 5:**
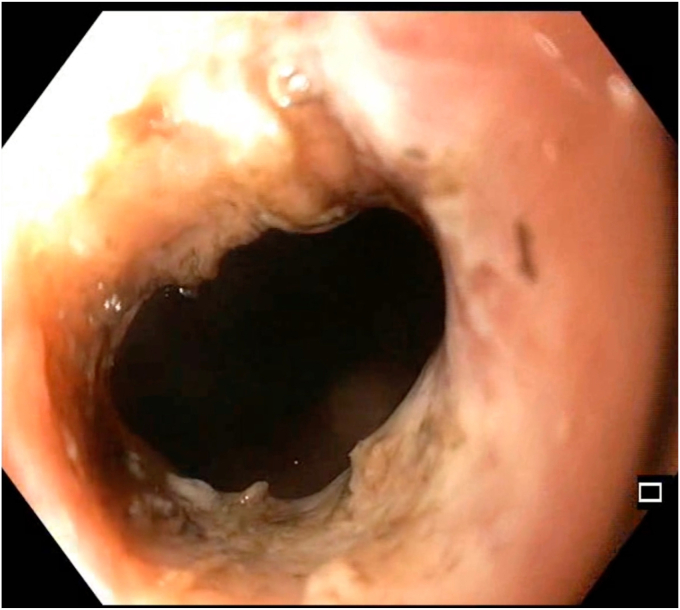
Ongoing dissection with extension of shallow circumferential incisions; segmental remodeling of the stricture wall allows lumen expansion without deep mural injury.

**Figure 6:**
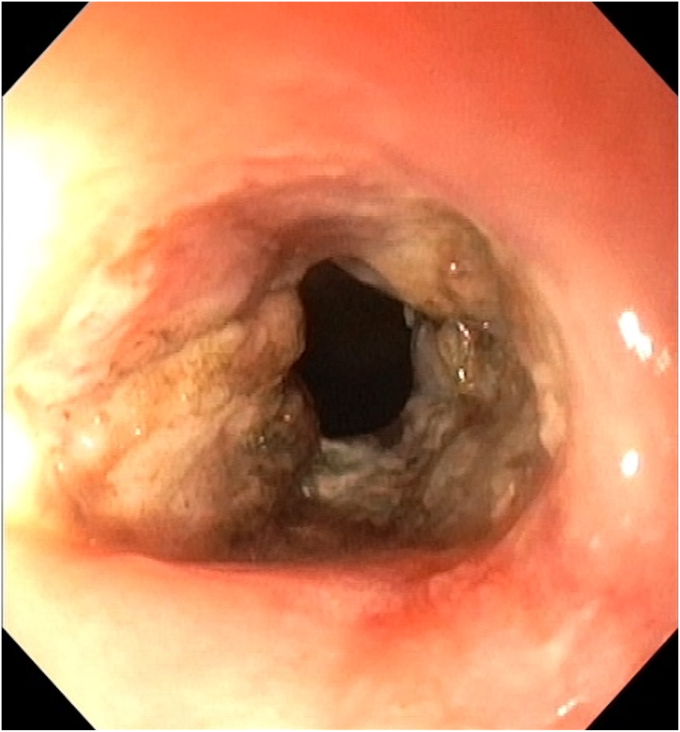
Dissection margins at third session showing circumferential release of fibrotic tissue displaying the actual extent of the stricture.

**Figure 7:**
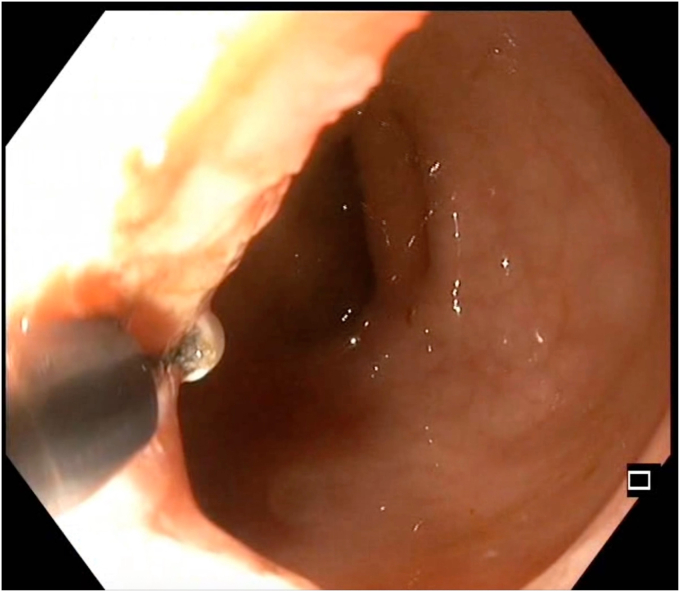
Endoscopic view showing the penultimate part of dissection.

**Figure 8:**
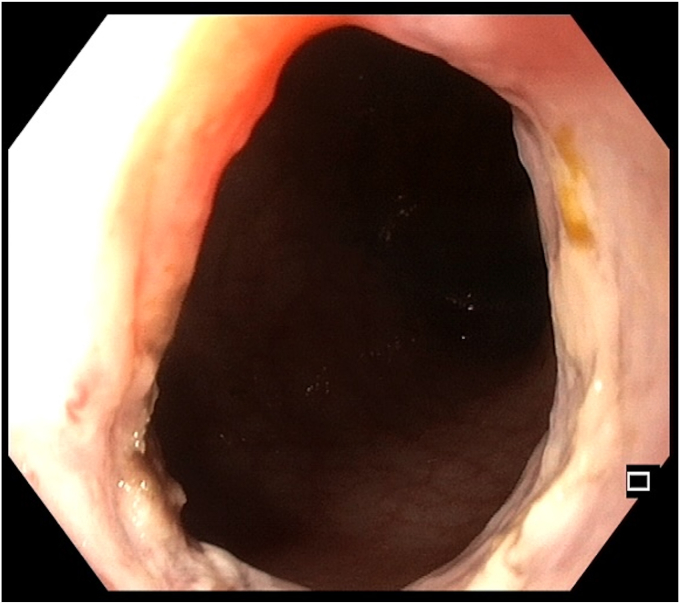
Endoscopic view showing widened lumen during last part of dissection.

An insulated-tip knife nano (Olympus) was used with ENDO CUT I mode (effect 3, duration 1, interval 3; VIO 300 D; Erbe Elektromedizin GmbH, Tübingen, Germany). A 15-mm SnareMaster (Olympus) was used to perform focal mucosectomy to expose the underlying stricture ring, facilitating subsequent layered dissection. SLICE was performed over 3 consecutive days, with gradual dissection of the fibrotic stricture performed using shallow radial incisions, layer by layer, in a circumferential manner (Figure 3, Figure 4, Figure 5, Figure 6, Figure 7, Figure 8, Video 1, available online at www.videogie.org). The Triangle Tip Knife (KD-640L; Olympus) and HookKnife (KD-620LR; Olympus) were used to dissect penultimate layers of redundant fibrotic tissue.

Prophylactic antibiotics were administered before each session. SLICE was performed over 3 consecutive days, with each session limited to 90 to 120 minutes. The first session was initiated in the evening and deferred because of limited time. The second session was halted early because of intraprocedural pain and distension, raising concern for a potential adverse event. Dissection was resumed after clinical reassessment. Visual safety cues included fibrotic ring delineation with the cap, tactile resistance, and avoidance of muscularis propria exposure.^1^ The patient was allowed only clear liquids between sessions and kept under inpatient observation. The total procedure time was approximately 5 hours across 3 settings. The stricture was traversed using a pediatric colonoscope at the end of the final session.

**Figure 9:**
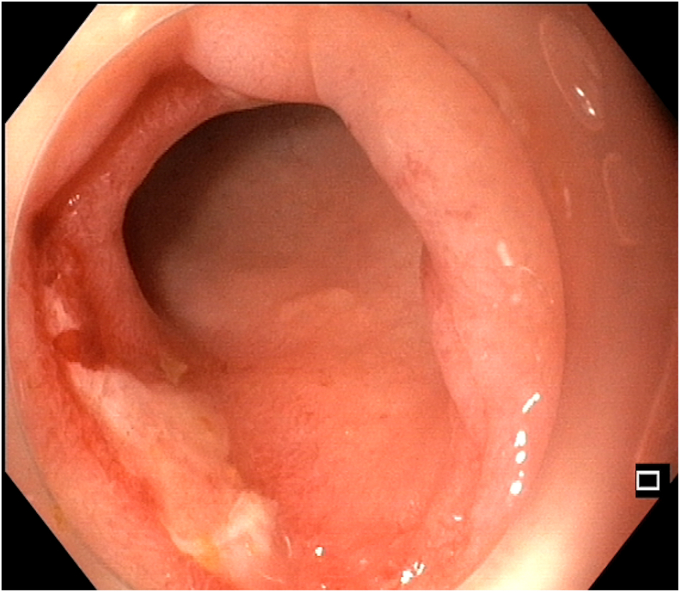
Follow-up endoscopic view at 6 months showing a patent lumen with only mild mucosal reapproximation.

At 6-month follow-up after completion of antitubercular therapy, intestinal ultrasound showed only mural thickening without luminal narrowing or prestenotic dilation (Video 1). Endoscopy showed mild mucosal reapproximation but a fully passable lumen without resistance (Fig. 9). A clip-based endoscopic stricturoplasty was performed at the final session as a day care procedure to prevent circumferential reclosure (Video 1).

SLICE offers advantages over radial incision and cutting (RIC), especially for fibrotic, angulated, or >1-cm strictures. Most knives used in stricturotomy have a cutting wire length ≤5 mm, making RIC technically challenging and less effective for long fibrotic strictures. SLICE, in contrast, uses staged layered incision and circumferential dissection along the entire length of the stricture to achieve controlled remodeling by adopting an endoscopic submucosal dissection–like approach.

Although endoscopic balloon dilation (EBD) remains a standard first-line approach, interim analysis of a randomized controlled trial from our group comparing endoscopic stricturotomy (ES) (using primarily the SLICE approach) and EBD in short Crohn’s disease strictures (<3 cm) showed significantly lower reintervention (15% vs 42.9%) and surgery rates (5% vs 28.6%) with stricturotomy, with comparable safety and no perforation.^2^ Functional luminal probe imaging data and previous retrospective studies in anastomotic strictures further support poor durability of EBD in fibrotic strictures due to elastic recoil.^3^^,^^4^ We did not use a lumen-apposing metal stent because of a high risk of migration and reintervention, lack of anchorage, and cost-equivalence to surgery.^5^^,^^6^ Hence, ES can be considered as a first-line or rescue therapy for short (<3 cm) strictures if expertise is available instead of EBD and a lumen-apposing metal stent. For strictures >1 cm, SLICE is a promising option as compared to RIC technique for ES.

In the event of a deep mural injury, we have successfully used hemostatic clips for closure in other cases (Video 1). Other rescue strategies are over-the-scope clips, suturing, and tattooing if surgery is needed.

SLICE and graded sessions are selectively reserved for complex strictures and not required for all cases.^7^ This builds on our previous work in hybrid and graded stricturotomy.^8^, ^9^, ^10^ In complex strictures, the safety and precision of SLICE help avoid adverse events like perforation/surgery/emergency laparotomy, which may outweigh the cost and time burden of staged intervention.

A recent cost-effectiveness analysis showed that stricturotomy strategies were more cost-effective than surgery.^11^ Future directions should include prospective studies comparing RIC versus SLICE techniques, standardization of safety checkpoints/procedure algorithms, and integration with preprocedural imaging. Additional follow-up data and reproducibility across centers are warranted to validate long-term utility.

## Patient consent

The patient in this article has given written informed consent to publication of their case details.

## Disclosure

The following author disclosed financial relationships: P. Pal: Consultant for Johnson & Johnson. All other authors disclosed no financial relationships.

## Supplementary data

Video 1 Stepwise demonstration of the segmental layered incision and circumferential endoscopic dissection (SLICE) technique for a long fibrotic stricture at the splenic flexure in a patient with suspected tubercular colitis. The procedure was performed over 3 consecutive sessions using a therapeutic endoscope with a transparent distal cap and an insulated-tip knife nano under Endocut I settings. Layered, shallow radial incisions were made in a circumferential manner, carefully confined to the fibrotic submucosal plane to avoid muscularis propria injury. Progressive remodeling of the stricture is demonstrated, culminating in complete luminal patency. Follow-up endoscopy at 6 months confirmed sustained lumen patency, which was treated with clip-based stricturoplasty as day care procedure to prevent reapproximation. The video also includes a schematic repersenatation of SLICE technique and troubleshooting segment from a separate case of an ascending colon stricture, where deep mural fibers were exposed after SLICE and successfully closed with hemoclips to prevent delayed perforation.
